# Altered metabolomic profiling of overweight and obese adolescents after combined training is associated with reduced insulin resistance

**DOI:** 10.1038/s41598-020-73943-y

**Published:** 2020-10-09

**Authors:** Renata G. Duft, Alex Castro, Ivan L. P. Bonfante, Wendell A. Lopes, Larissa R. da Silva, Mara P. T. Chacon-Mikahil, Neiva Leite, Cláudia R. Cavaglieri

**Affiliations:** 1 Laboratory of Exercise Physiology, Faculty of Physical Education, University of Campinas (UNICAMP), Av. ÉricoVeríssimo, 701, Campinas, São Paulo Brazil; 2 Department of Physical Education, State University of Maringa, Maringa, Brazil; 3 Department of Physical Education, University of Parana, Curitiba, Brazil

**Keywords:** Biomarkers, Metabolomics

## Abstract

Exercise training and a healthy diet are the main non-pharmacological strategies for treating chronic conditions, such as obesity and insulin resistance (IR), in adolescents. However, the isolated metabolic changes caused by exercise training without dietary intervention have not yet been established. We investigated how combined training (CT) without dietary intervention altered the concentrations of serum metabolites, biochemical, anthropometric and functional parameters in overweight and obese adolescents. Thirty-seven adolescents (14.6 ± 1.05 years), of both sexes, were randomly assigned to the control group (CG, n = 19) or the training group (TG, n = 18). The CT was composed by resistance training and aerobic training performed in the same session (~ 60 min), three times a week, for 12 weeks. All assessments were performed pre and post-intervention. Metabolomics analyses were conducted using nuclear magnetic resonance spectroscopy (^1^H NMR) in a 600 MHz spectrometer. There was a decrease in body weight (BW), body mass index (BMI), waist circumference (WC), % body fat (%BF), fasting glucose, insulin levels, and insulin resistance (IR), by HOMA-IR, in the TG. An increase in fat-free mass (FFM) was also observed in the CG. The metabolic changes were given mainly by changes in the levels of metabolites 2-oxoisocaproate (↓TG), 3-hydroxyisobutyrate (↑CG and ↓TG), glucose (↓TG), glutamine (↓CG and ↑TG) and pyruvate (↓TG). These findings demonstrate the positive effects of CT program without dietary intervention on metabolomic profile, body composition, biochemical markers, and glucose metabolism in overweight and obese adolescents.

## Introduction

Obesity in childhood and adolescence has been considered one of the most serious public health problems in the world^1^. A sedentary lifestyle together with unhealthy eating habits leads to the onset of noncommunicable diseases (NCDs), such as type 2 diabetes (T2D), cardiovascular diseases (CVDs), and obesity^2^. Despite being a multifactorial disease, the mechanisms of the systemic energy imbalance underlying obesity are well explained^3^. However, the contribution of unhealthy lifestyle factors to the progression of obesity, along with optimal non-pharmacological treatment strategies, remains to be elucidated.

In order to explore a broad biological view of metabolism, Systems Biology has been largely used in the recent years. The metabolomics approach identifies and quantifies metabolites, which are key products to understand the metabolic state of an organism^4^. The NMR biofluid has proven itself to be extremely useful in biomarker discoveries over the years^5, 6^. A set of metabolites, under a physiological condition, is called a metabolome, which is affected by disorders in metabolic homeostasis, such as genetic perturbations, nutrition, obesity, and physical exercise^7^.

Exercise training is usually recommended as non-pharmacological therapy for the prevention and treatment of obesity and associated comorbidities^8^. The association of obesity with physical inactivity, in children and adolescents, can lead to many risks of cardiovascular and metabolic diseases in adult life^9^. The combination of resistance training (RT) with aerobic training (AT), also known as combined training (CT), seems to be a useful strategy to change body composition, and to reduce biochemical and inflammatory markers in obese adolescents^10, 11^, improving the overall health in this population^12, 13^.

A recent review provided an update of the publications using metabolomics in studies with different types of physical exercises^14^. However, all of the reviewed studies analyzed the effects of exercise training on the metabolic response in apparently healthy adults. Furthermore, as far as we know, previous studies have not investigated the metabolic responses to combined training in obese adolescents, using a metabolomic approach. Besides, previous studies included dietary interventions in addition to exercise training program, which made it impossible to understand only the effects of physical training alone on the metabolism of obese adolescents regardless of their diet, which reflects the real-life scenario^15–17^.

Therefore, although the beneficial effects of physical exercise for children and adolescents, such as changes in body composition and improvement of cardiorespiratory fitness, strength and chronic inflammation, are well-known, there is still no data in literature elucidating the global metabolic changes promoted by a exercise training program in obese adolescents. Thus, this study aimed primarily to analyze the changes in the metabolomic profile of overweight and obese adolescents after 12 weeks of CT without dietary intervention. Secondarily, we investigated changes in body composition, physical fitness, biochemical markers and insulin resistance (IR) in this population.

## Results

### Biochemical, anthropometry, body composition, and dietary patterns

The comparison between the CG and the TG (Pre vs. Post) presented some significant differences (Table 1). Fasting glucose levels showed main effect of time (p = 0.010), while insulin levels (p = 0.027) and insulin resistance, by HOMA-IR (p = 0.015) decreased in the TG, with no changes observed for the CG (p > 0.05 for all). The total cholesterol levels did not change in either groups (p = 0.951).

**Table 1:** General characteristics of the CG and the TG pre and post-intervention.

Variable	CG (n = 19)		TG (n = 18)		p-value
	Pre	Post	Pre	Post	
Sex ratio	48% Boys/52% Girls		50% Boys/50% of girls		0.632
Age (years)	14.72 ± 1.07		14.44 ± 1.04		0.476
BW (kg)^b^	78.77 ± 9.40	79.78 ± 10.27*	78.20 ± 8.88	77.14 ± 8.70*	0.004
Height (m)^b^	1.63 ± 0.07	1.65 ± 0.07*	1.65 ±0.06	1.66 ± 0.07	0.007
BMI (kg/m^2^)^b^	29.53 ± 2.80	29.82 ± 2.95	28.34 ± 2.33	27.93 ± 2.48*	0.013
BMI z-score^b^	2.08 ± 0.48	2.04 ± 0.48	2.18 ± 0.50	2.06 ± 0.52*	0.011
WC (cm)^b^	91.14 ± 6.63	92.99 ± 7.14*	92.14 ± 7.64	89.29 ± 7.46*	< 0.001
%BF^b^	41.66 ± 5.45	42.99 ± 5.78*	40.32 ± 5.53	37.78 ± 5.55*	< 0.001
FFM (%)^b^	53.48 ± 7.17	53.41 ± 6.67	56.77 ± 7.05	59.21 ± 7.76*	0.004
Fasting glucose (mg/dL)^a^	88.22 ± 8.22	85.63 ± 8.38	86.61 ± 8.30	82.02 ± 7.36*	0.010
Insulin (mcUI/mL)^b^	15.07 ± 7.86	17.44 ± 6.76	16.47 ± 8.96	13.81 ± 7.63*	0.027
HOMA-IR^b^	3.33 ± 1.88	3.67 ± 1.48	3.48 ± 1.72	2.81 ± 1.57*	0.011
Total Cholesterol (mg/dL)	170.67 ± 33.91	170.24 ± 35.02	166.91 ± 35.20	166.89 ± 34.24	0.951
1RM—leg press (kg)^b^	172.50 ± 30.48	181.25 ± 33.58	170.83 ± 44.95	224.06 ± 53.61*	< 0.001
1RM—bench press (kg)^b^	32.18 ± 4.35	33.00 ± 3.61	32.33 ± 6.41	43.40 ± 8.86*	< 0.001
VO_2peak_ (mL/kg/min)^b^	35.18 ± 6.22	35.06 ± 5.50	32.80 ± 6.15	37.66 ± 6.19*	< 0.001

Data are mean ± SD.

*CG* control group, *TG* training group, *BW* body weight, *BMI* body mass index, *WC* waist circumference, *%BF* percentage of body fat, *FFM* fat-free mass, *HOMA-IR* homeostatic model assessment, *WC* waist circumference.

*Significant difference from pre moment in post hoc analysis (p < 0.05).

^a^Significant ANOVA main effect of time (p < 0.05).

^b^Significant ANOVA group*time interaction (p < 0.05).

The variables BW (p = 0.004), WC (p < 0.001), BMI (p = 0.013), %BF (p < 0.001), FFM (p = 0.004), maximal strength on the leg press (p < 0.001), bench press (p < 0.001), and VO_2peak_ (p < 0.001) had interaction group*time in the ANOVA analysis. The CG increased BW (p = 0.034) and WC (p = 0.039), while a reduction was observed of BW (p = 0.039), WC (p = 0.002) and BMI (p = 0.032) in the TG. Regarding body composition, the %BF decreased (p < 0.001) in the TG and increased in the CG (p = 0.018). For the physical fitness variables, only the TG presented an increase in the maximal strength on leg press (p < 0.001), bench press (p < 0.001) and VO_2peak_ (p < 0.001) after 12 weeks of intervention (p < 0.001). There were no significant differences in total energy intake and macronutrient consumption within or between the CG and the TG pre and post-intervention (p > 0.05 for all) (Table 2).

**Table 2:** Dietary intake in the CG and the TG pre and post-intervention.

Variable	CG (N = 19)		TG (N = 18)		p-value
	Pre	Post	Pre	Post	
Total energy intake (kcal)	2,383.4 ± 808.9	2,409.9 ± 762.7	2,262.0 ± 515.9	2,134.2 ± 296.4	0.603
**Carbohydrates**					
(g)	298.1 ± 78.2	302.0 ± 125.4	298.8 ± 75.4	282.5 ± 46.2	0.618
(%)	52.4 ± 6.9	52.6 ± 5.9	52.6 ± 10.5	53.4 ± 8.5	0.874
**Proteins**					
(g)	96.5 ± 33.0	98.4 ± 27.5	92.2 ± 23.8	88.4 ± 20.0	0.647
(%)	16.7 ± 3.2	17.3 ± 2.3	16.5 ± 2.7	16.6 ± 2.9	0.702
**Lipids**					
(g)	78.0 ± 37.5	77.8 ± 36.3	65.8 ± 19.6	77.6 ± 22.9	0.398
(%)	28.8 ± 7.7	28.6 ± 8.7	26.9 ± 6.5	33.2 ± 10.3	0.101

Data are mean ± SD.

*CG* control group, *TG* training group.

### Metabolic changes by training

**Figure 1:**
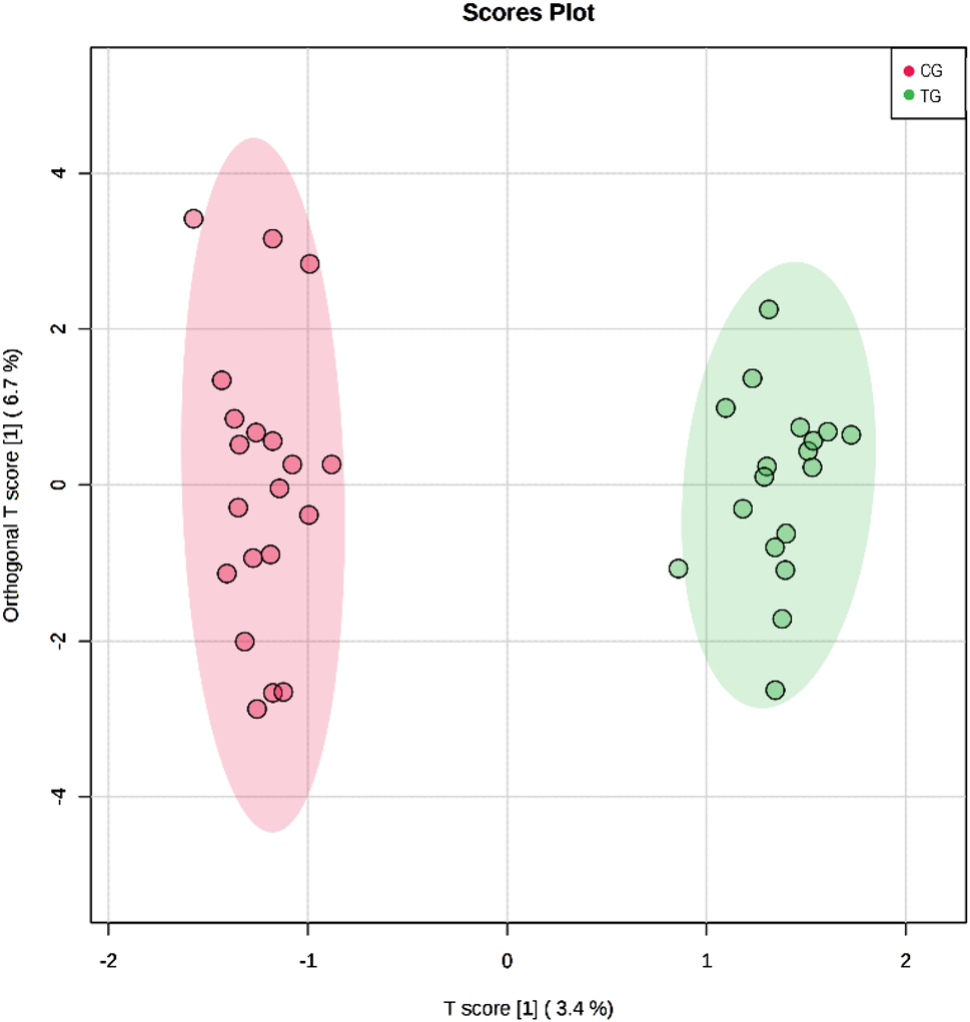
Orthogonal partial least square discriminant analysis (OPLS-DA) within the control group (CG) represented in red, and the training group (TG) in green, after 12 weeks of combined training. The model was validated with permutation tests (one hundred permutations p < 0.01) and cross-validation (R2Y = 0.986 and Q2 = 0.54).

**Figure 2:**
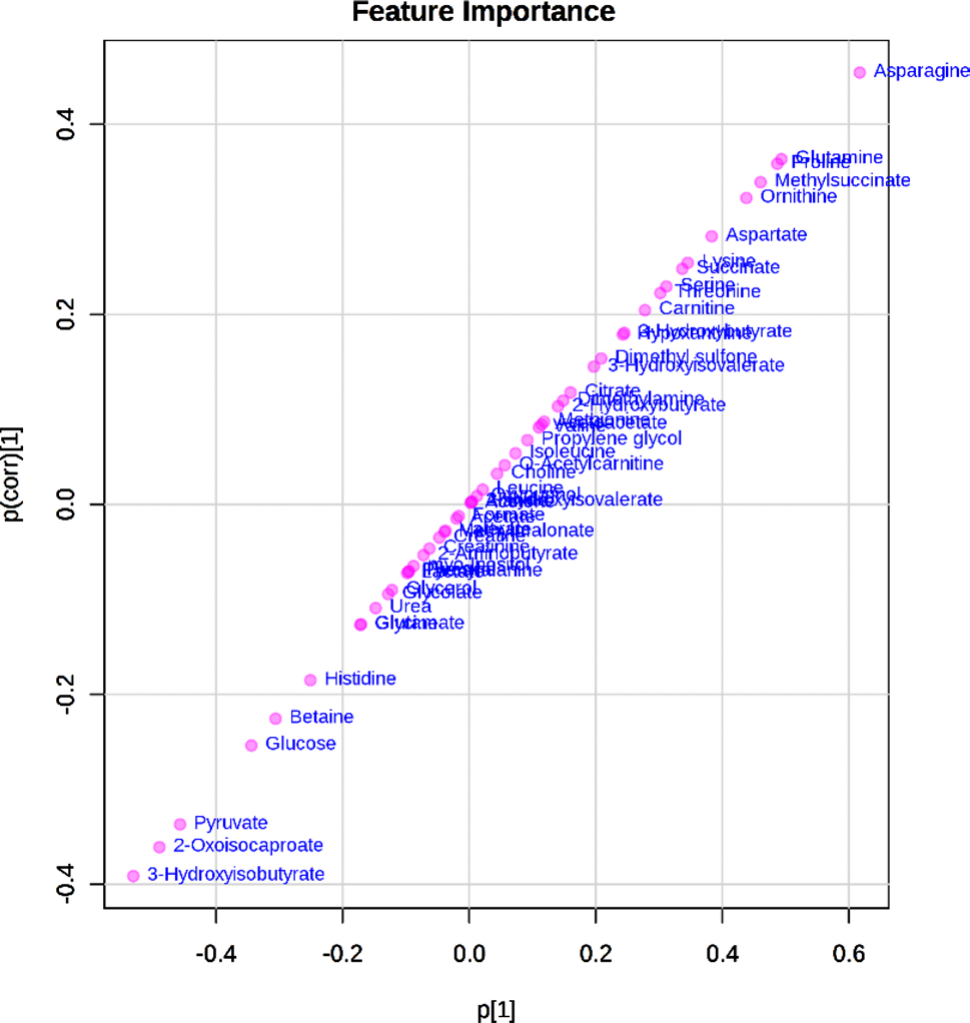
S-plot of the metabolites differentiators between the control group (CG) and the training group (TG) after 12 weeks of intervention, with signals on the upper right end being related to changes in the control group, and signals on the lower left end being related to changes in the TG.

A total of 51 metabolites were identified and quantified. Figure 1 shows the segregation between groups through the scores plot OPLS-DA graph, and Fig. 2 shows the S-PLOT graph with the main metabolites responsible for the group’s segregation. The 20 main metabolites selected from S-PLOT and their concentrations are presented in Table 3. Five metabolites showed significant interaction group*time in ANOVA analysis: 2-oxoisocaproate (p = 0.028), 3-hydroxyisobutyrate (3-HIB) (p = 0.031), glucose (p = 0.014), glutamine (p = 0.004), and pyruvate (p = 0.038). Among them, 2-oxoisocaproate, 3-HIB, glucose, and pyruvate had their concentration decreased in the TG, while glutamine increased its levels after 12 weeks of CT (Table 3). In the CG, the concentrations of 3-HIB significantly increased, while glutamine and proline decreased.

**Table 3:** Concentration of the main metabolites selected by S-PLOT. Data are mean ± SD.

Metabolite (mM)	CG (N = 19)		TG (N = 18)		p-value
	Pre	Post	Pre	Post	
2-Oxoisocaproate^b^	0.0016 ± 0.0003	0.0016 ± 0.00048	0.0019 ± 0.0080	0.0016 ± 0.0005*	0.028
3-Hydroxyisobutyrate^b^	0.0052 ± 0.0016	0.0061 ± 0.0021*	0.0054 ± 0.0013	0.0046 ± 0.0015*	0.031
Asparagine	0.0181 ± 0.0033	0.0175 ± 0.0034	0.0174 ± 0.0023	0.0179 ± 0.0031	0.246
Aspartate	0.0134 ± 0.0030	0.0126 ± 0.0027	0.0128 ± 0.0023	0.0136 ± 0.0020	0.128
Betaine	0.0088 ± 0.0028	0.0088 ± 0.0039	0.0079 ± 0.0027	0.0070 ± 0.0029	0.343
Carnitine	0.0119 ± 0.0030	0.0112 ± 0.0031	0.0114 ± 0.0025	0.0112 ± 0.0025	0.497
Glucose^a^	1.4178 ± 0.1510	1.3852 ± 0.2054	1.4749 ± 0.1424	1.3650 ± 0.1307*	0.014
Glutamine^b^	0.1542 ± 0.0199	0.1376 ± 0.0295*	0.1376 ± 0.0295	0.1494 ± 0.0209*	0.004
Lysine	0.0477 ± 0.0098	0.0471 ± 0.0105	0.0466 ± 0.0060	0.0448 ± 0.0066	0.665
Methylsuccinate	0.0018 ± 0.0003	0.0017 ± 0.0005	0.0018 ± 0.0008	0.0018 ± 0.0004	0.828
Ornithine^a^	0.0183 ± 0.0046	0.0187 ± 0.0043	0.0146 ± 0.0044	0.0159 ± 0.0038	0.493
Proline^b^	0.0928 ± 0.0286	0.0765 ± 0.0225*	0.0864 ± 0.0163	0.0826 ± 0.0162	0.040
Pyruvate^b^	0.0175 ± 0.0092	0.0193 ± 0.0078	0.0239 ± 0.0107	0.0185 ± 0.0096*	0.038
Serine	0.0471 ± 0.0080	0.0442 ± 0.0097	0.0442 ± 0.0097	0.0459 ± 0.0061	0.186
Succinate	0.0027 ± 0.0008	0.0024 ± 0.0006	0.0028 ± 0.0006	0.0030 ± 0.0008	0.061
Threonine	0.0588 ± 0.0136	0.0564 ± 0.0154	0.0561 ± 0.0138	0.0552 ± 0.0088	0.697

*CG* control group, *TG* training group.

*Significant difference from PRE in post hoc analysis (p < 0.05) after the adjustment of Benjamini–Hochberg false discovery rate.

^a^Comparisons between and within groups analyzed by ANCOVA (adjusted data by pre-values).

^b^Significant ANOVA group*time interaction (p < 0.05).

In relation to the TG, an association between changes in 3-HIB concentrations and VO_2peak_ (r = 0.566, p < 0.001) was observed, as well as an association between changes in pyruvate and weight (r = 0.589, p < 0.001), BMI (r = 0.598, p < 0.01) and WC (r = 0.641, p < 0.01).

## Discussion

The purpose of this study was to analyze the changes in the metabolomic profile of overweight and obese adolescents after 12 weeks of CT without dietary intervention. Also, we investigated changes in body composition, physical fitness, biochemical markers and insulin resistance (IR) in this population. The main findings of the present study were the changes in the concentration of serum metabolites related to glucose metabolism and IR such as 2-oxoisocaproate, 3-hydroxyisobutyrate, glucose, glutamine, pyruvate, after CT, as well as significant improvement of body composition, physical fitness and insulin resistance (IR) in overweight and obese adolescents. These finding not only reinforce the effectiveness of CT in improving factors associated with obesity, but also point out some candidates metabolites that would be related to the improvement in glucose metabolism and IR promoted by CT in this population.

Regarding metabolic changes, the metabolite 2-oxoisocaproate (α-ketoisocaproic acid—KIC) is a product of leucine (Leu) transamination catalyzed by the enzyme BCAT (branched-chain amino-acid aminotransferase) catabolism, which can be interconverted to leucine or follow two pathways^12^. In the first pathway, in the mitochondria, oxidative decarboxylation of KIC occurs, catalyzed by the branched-chain α-keto acid dehydrogenase (BCKDH) complex, resulting in acetoacetate and acetyl-CoA^18, 19^. The second path occurs in peripheral tissues and the liver, oxidizing KIC in the cytosol to 3-hydroxy-3-methylbutyrate (HMB), which has ergogenic benefits such as an increase in FFM^20^, as observed in Table 1.

Obese individuals have higher concentrations of BCAAs and, consequently, the products of their degradation^21^. Some studies have shown that high levels of BCAAs in the bloodstream are associated with obesity and IR^22–24^. However, BCAAs oxidation and their catabolic products increase during physical exercise^25^. There was a decrease in the concentration of this metabolite after the CT, probably because 2-oxoisocaproate was used in the protein synthesis to increase FFM, and the circulating levels were reduced. Additionally, IR also decreased, highlighting exercise training as a key tool to reduce the levels of BCAAs in obese individuals.

**Figure 3:**
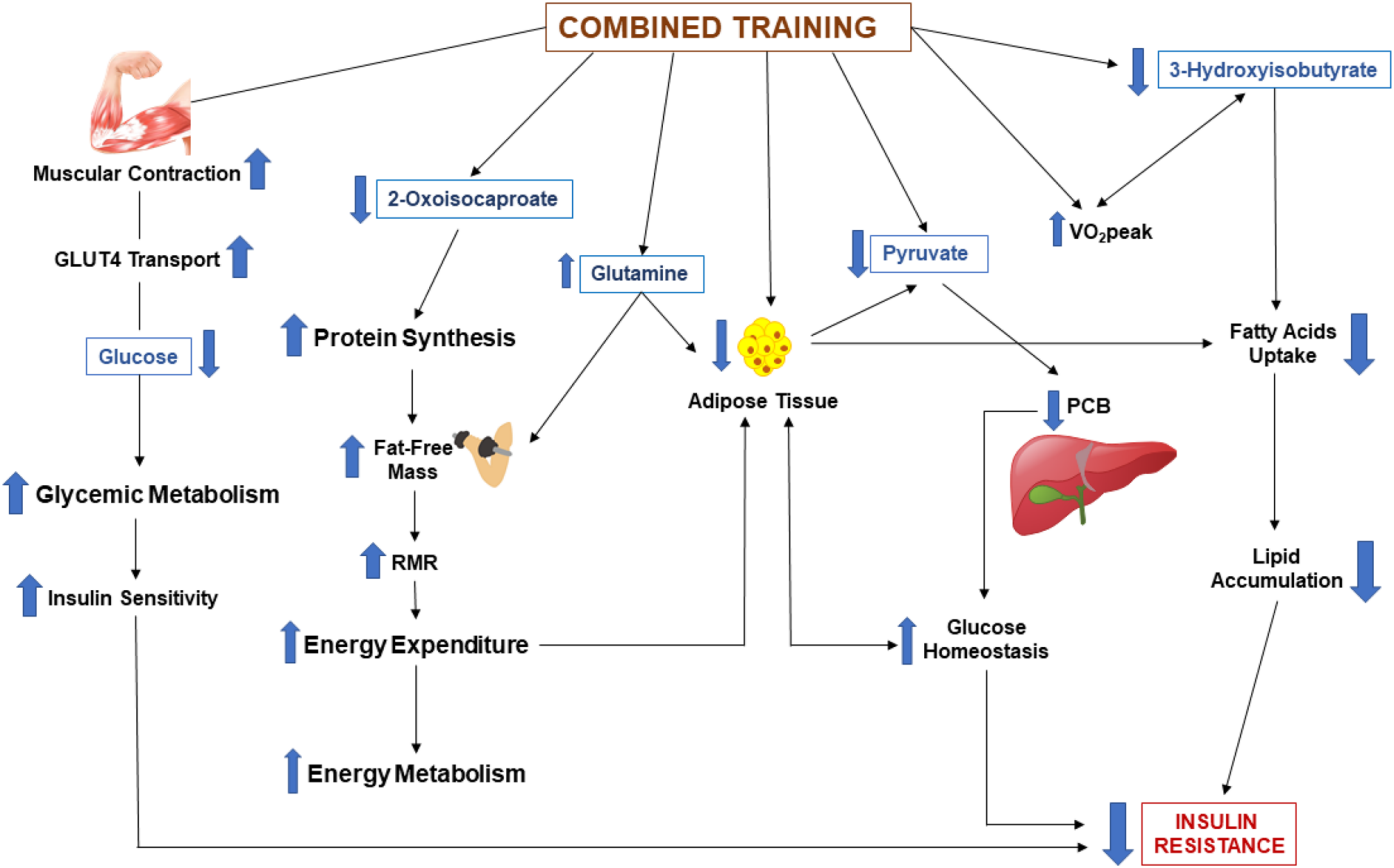
Main findings and possible metabolic relationships. Combined Training (CT) increases muscular contraction and the translocation of glucose into the cells via GLUT-4, consequently decreasing glucose levels in the bloodstream. CT decreases the levels of 2-oxoisocaproate to use it in protein synthesis to increase fat-free mass (FFM), increasing rest metabolic rate (RMR), energy expenditure, and energy metabolism. The increase in energy expenditure leads to the reduction of adipose tissue (%BF), the improvement of glucose homeostasis, and the consequent reduction of insulin resistance (IR). The training also decreases the %BF, improving pyruvate metabolism, reducing pyruvate levels, and pyruvate carboxylase, which also improves glucose homeostasis. The increase in glutamine levels leads to an increase FFM and a decrease in %BF. Finally, a decrease in 3-hydroxyisobutyrate levels occurrs, decreasing fatty acids uptake, leading to a decrease in lipid accumulation and improving IR.

The metabolite 3-HIB is derived from valine metabolism and was recently associated with the incidence of T2D^26^. It was also shown that secreted 3-HIB enhances fatty acids uptake, leading to an increase of lipid accumulation and induction of IR^26^. The plasma levels of 3-HIB were strongly influenced by the amount of adipose tissue, and it is believed that 3-HIB could be considered a future marker of T2D risk, as well as an important factor for the regulation of metabolic flexibility^26^. Our results showed an increase in the levels of 3-HIB in the CG and a reduction of its concentration in the TG. The HOMA-IR and the amount of %BF also decreased, corroborating the study previously mentioned^26^. The metabolite 3-HIB was modulated by the exercise training, which can also be observed in the association between the changes in 3-HIB concentrations and VO_2peak_. Thus, the metabolite 3-HIB could be an interesting biomarker to explore in the prevention of comorbidities as T2D (Fig. 3).

Another metabolite that showed a decrease after the CT was glucose, which was expected since the mechanisms underlying muscle contraction and glucose transporter type 4 (GLUT-4) are well established in the literature^27^. However, an important finding to point out is that the CT promoted a reduction of the levels of insulin and HOMA-IR, leading to a decrease in IR. This result might be related to an improvement in the sensitivity of the insulin receptor due to the anti-inflammatory effect of exercise, against the pro-inflammatory consequence of obesity^28^. This anti-inflammatory effects of CT are not presented in this study, but have already been reported by Lopes^11^.

Glutamine (Gln), the most abundant non-essential amino acid in human plasma, is synthesized in the skeletal muscle and liver cells, and released into blood circulation. It is necessary mainly for energy production, gut barrier function, immune function, nucleic acid synthesis, acid–base balance, and glutathione (GSH) production^29^. A link has already been shown, in literature, between circulating levels of glutamine, body weight, and metabolism, where low glutamine levels predict the incident of IR and T2D^30^. A recent study discovered that the amino acid Gln was the most markedly reduced factor in obese subjects. The study combined studies in human and mice cell cultures, and could observe that glutamine rewired the metabolism of adipose tissue cells and lowered the expression of inflammatory genes^31^. A study presented a systematic review and meta-analysis on the effects of Gln supplementation on health status^32^. Among the benefits are the improvement of glucose-stimulated insulin secretion, the decrease in IR, fasting glucose levels and body fat, and the increase in fat-free mass, which may consequently help reduce obesity, T2D, and IR^32^.

The anti-inflammatory effects of exercise are well-established in literature^8, 33^, and also the anti-inflammatory effect of CT in adolescents^10, 11^. Although our subjects did not supplement Gln, an increase in its levels was observed after 12 weeks of CT. At the same time, there was a decrease in the %BF and an increase in FFM, as well as a decrease in IR. Besides, this increase in glutamine levels through exercise could be associated with the changes previously mentioned. Glutamine should be further investigated in studies with exercise training programs in obese and individuals with T2D.

The last metabolite, pyruvate, is an α-keto acid present in multiple biochemical pathways intersections. It is usually an end product of glycolysis which, under aerobic conditions, is transported to the mitochondria and converted to acetyl-CoA for entering the TCA cycle, in order to produce energy (ATP)^34^. This metabolite can also be involved in the anabolic synthesis of fatty acids and amino acids, such as alanine, or converted to ethanol and lactic acid by fermentation^35^. The study by Pechlivanis^36^ found a decrease in pyruvate concentrations after 8 weeks of aerobic training. One of the explanations for this was the removal of lactate and pyruvate from the bloodstream, promoted by the exercise training. Another recent study showed the role of physical exercise and pyruvate in hepatic glucose production. For the maintenance of glycemic homeostasis, the production of hepatic glucose is essential. However, studies have shown the importance of its suppression through the activity of the pyruvate carboxylase (PCB) enzyme, which converts pyruvate to oxaloacetate in the mitochondria in the first part of the gluconeogenic pathway^37, 38^. The control of glucose homeostasis through PCB has been proven by its inhibition, decreasing blood glucose, attenuating hepatic glucose production, and reducing the development of hepatic steatosis in animal models^39^. The role of exercise training in improving insulin sensitivity, reducing the amount of adipose tissue and hepatic glucose, and improving glucose homeostasis, is already known in literature^40^. It was showed that exercise training reduced hepatic PCB, leading to improved fasting glucose, insulin, and IR in mice^37^. Our findings may corroborate this the ones in this study, since we had a reduction of pyruvate levels, fasting glucose, insulin, and IR. Probably the same mechanism found in mice could be present in humans (Fig. 3). However, we cannot assure that, since we did not measure fructose 1,6 bisphosphatase, a key enzyme in the gluconeogenesis pathway.

Another interesting point related to the changes in pyruvate is that the amount of adipose tissue can cause a disorder in pyruvate metabolism, because of the impaired regulation of carbohydrate metabolism, leading to metabolic inflexibility^12^. Metabolic inflexibility is defined as the inability of switching from fatty acids to glucose oxidation, increasing lipolysis in patients with IR, obesity, or T2D^41^. The levels of pyruvate in the CG were higher than in the TG, probably because of the amount of adipose tissue (Table 3). After the intervention, %BF was significantly reduced in the TG, leading to an improvement in pyruvate metabolism and glucose homeostasis (Fig. 3). We also observed an association between changes in pyruvate and changes in BW, BMI, and WC after 12 weeks of CT, suggesting that the changes in these variables related to body composition are associated with pyruvate changes promoted by exercise training.

This study has some limitation that should be mentioned. First, we used HOMA-IR to assess IR, which is less accurate than hyperinsulinemic-euglycemic clamp. Second, the sample size used in this study was small, but still presented statistical power, and included overweight and obese adolescents, which may have contributed to the absence of effects on some metabolic markers. However, it was composed only by adolescents, with similar pubertal stages, which reduced the possible effects puberty on these markers. Finnaly, we did not control the girls' menstrual cycle. This hormonal oscillation that occurs during the month in girls might cause some changes in the metabolic profile. However, 75% of the girls were in the same phase of the menstrual cycle and none of them were on their periods, which reduced the chances of hormonal oscillation influences.

This study was the first one to use the metabolomics approach to analyze the effects of 12 weeks of CT without dietary intervention in obese adolescents, with strict training loads control and high attendance rate. In summary, there were significant differences in anthropometric measures, such as height (↑), weight (↓), BMI (↓) and WC (↓), between groups. %BF (↓) and FFM (↑) were also significantly different between groups (Table 1). In relation to biochemical markers, there were significant differences in fasting glucose (↓), insulin (↓), and HOMA-IR (↓). Furthermore, we observed metabolic changes in metabolism after 12 weeks of combined training, having 2-oxoisocaproate (↓), 3-HIB (↓), glucose (↓), glutamine (↑) and pyruvate (↓) as the main changes (Table 3, Figs. 1, 2 and 3).

## Conclusion

The combined training without diet intervention promoted a reduction of body weight, waist circumference, fat mass, and the improvement of VO_2peak_, which are essential to reduce the risk of developing CVDs, as well as the improvement of fasting glucose and insulin, which are important to prevent the onset of T2D. These benefits may also be related to changes in the metabolomic profile of these overweight and obese adolescents, which were evidenced by the revealed serum markers. Among the main results and associations, there was a decrease in adipose tissue, an improvement of glycemic metabolism, leading to a decreased of insulin resistance. Therefore, combined training seems to be a good option of exercise training for overweight and obese adolescents in order to reduce the risk of developing obesity-related comorbidities, regardless of dietary intervention.

## Material and methods

All the procedures described in this manuscript were approved by the Ethics Committee of the Clinical Hospital of the Federal University of Parana (CAAE: 0063.0.208.000-11), and this study was registered in the Brazilian Clinical Trials Registry (RBR-35jq4c-28/03/2018), where the full trial can be accessed. The procedures described in this article were conducted according to the Helsinki Declaration^42^. The adolescents and their parent(s)/or legal guardian(s) signed the informed consent term and assent term, respectively. This study is part of another project, and other results were already published^11^.

### Subjects

This study is a parallel experimental study, composed of adolescents, of both sexes, aged 13–17 years, from the 8th grade of elementary school to the 3rd grade of high school, of a public school. The inclusion criteria were: having a BMI z-score ≥  + 1; being in the pubertal stage 4 or 5 (Tanner’s stages); having availability to participate in assessments and/or interventions; having the consent of their parent(s) or legal guardian(s) and agreeing on participating in the study. The exclusion criteria included: the presence of diabetes type 1 or 2; previous diagnoses of dyslipidemia; hypo/hyperthyroidism; pubertal delay; growth hormone deficiency; severe asthma; the presence of acute or chronic inflammatory disease; being on a diet or using dietary supplements regularly; participating in regular physical activity programs other than physical education at school, and having less than 80% of attendance on training sessions.

**Figure 4:**
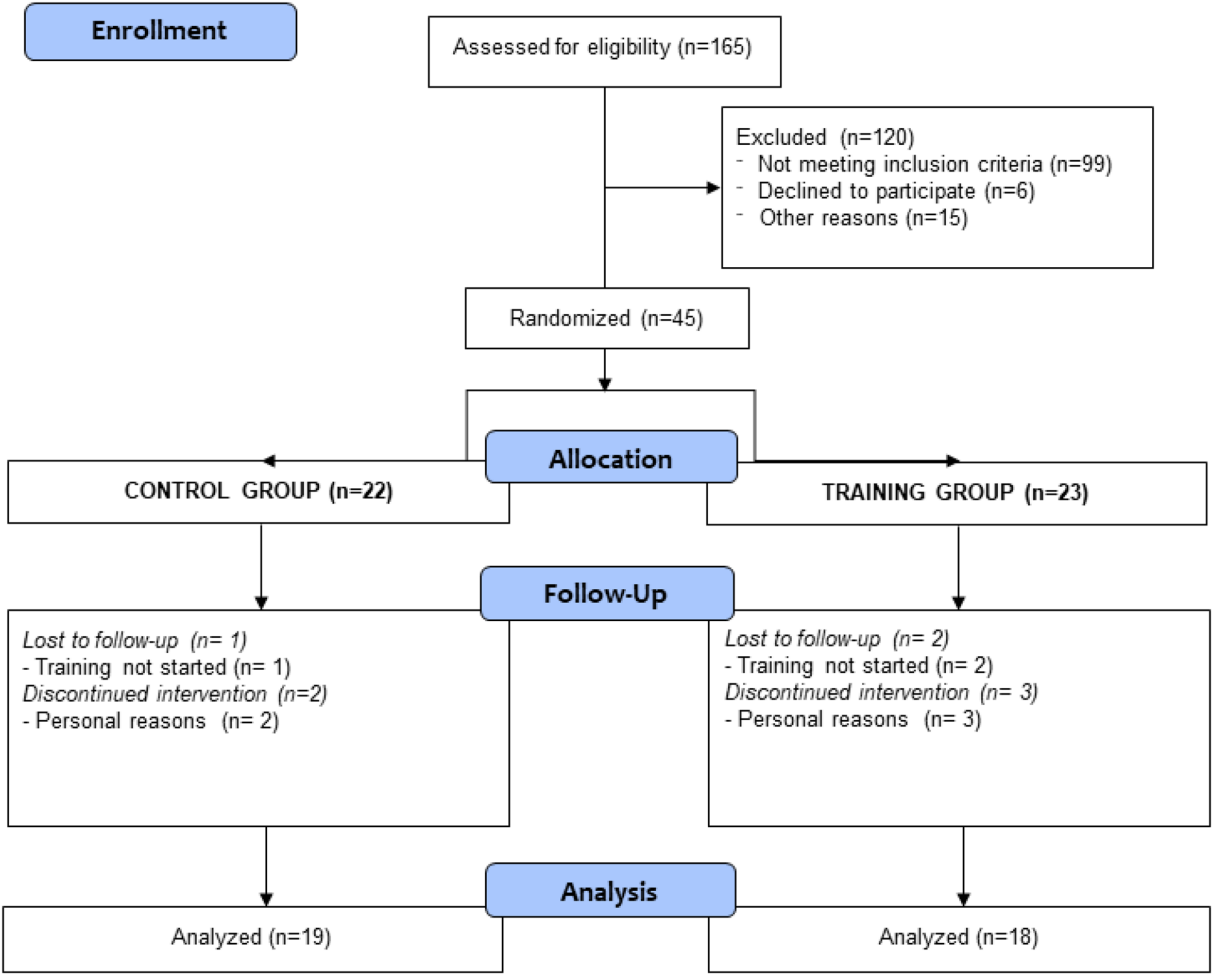
CONSORT flow diagram.

Initially, 165 adolescents were considered eligible for the study. After the application of the inclusion criteria, 45 adolescents were enrolled in the study. They were allocated randomly into the control group (CG) or the training group (TG). Three adolescents dropped out of the CG, and five of the TG, totalizing 37 subjects at the end of the study (CG = 19 and TG = 18) (Fig. 4).

The randomization number sequence was created using Excel 2019 (Microsoft, Redmond, WA, USA) with a 1:1 ratio allocation. The randomization was performed by a researcher not involved in this project to avoid selection bias. The sample size was calculated based on univariate and multivariate designs. Firstly, the sample size was calculated a priori using the GPower 3.1 software, based on generic moderate effect sizes (Cohen’s ƒ = 0.3) of previously published studies^11, 12, 43^, assuming a within-between design (group*moment interaction), Type I error rate (α) of 5% for a two side-test and correlation among repeated measures of r = 0.5 to ensure at least 80% of statistical power (1 − β) in the univariate analyses^12^. Secondly, a post hoc sample statistical power analysis was calculated based on a multivariate design using the MetaboAnalyst 4.0 software. Then, assuming a false discovery rate of 0.2 and a mean effect size estimated from all metabolites retained after multivariate analysis, we have confirmed a statistical power of at least 70% in between-group comparisons.

### Anthropometric, body composition and pubertal stage assessments

Body weight (BW) and height were measured using a calibrated standard scale (Filizola, São Paulo, Brazil) with resolution of 0.1 kg and accuracy of 0.1 cm. Body mass index (BMI) was calculated by the formula: [weight (kg)/(height (m))^2^]. BMI z-score was calculated and classified according to criteria defined by the World Health Organization growth charts for sex and age^44^. Waist circumference (WC) was measured at the midpoint between the last ribs and the iliac crests, with an inelastic measuring tape, with the precision of 0.1 cm, being the participant in a standing position, and having overnight fasted^44^. The measurement was performed in triplicate by a single trained professional, and the average of these three measurements was calculated. Dual X-ray absorptiometry assessed body composition in Lunar Prodigy Primo (General Electric Healthcare; Madison, WI), according to Rodriguez et al.^45^.

The pubertal evaluation was conducted by a pediatric endocrinologist, according to the development of breasts (B) in girls, genitals (G) in boys, and pubic hair (P) in both, using the method by Marshall and Tanner^46, 47^. Breast and genitals were examined according to size, shape, and characteristics and pubic hair, according to quantity and distribution^48^. The Tanner’s stages 4 and 5 were considered when at least one of the secondary sexual characteristics (B and P for girls and G and P for boys) reached stages 4 or 5. In the absence of 4 or 5 for these secondary sexual characteristics, the participant was not included in the study.

### Blood collection and biochemical analysis

Blood samples were collected from the antecubital vein in dry tubes (8 mL), Vacutainer brand (Becton Dickinson Ltd, Oxford, UK), after a 12-h overnight fast. The samples were obtained before the beginning of the intervention, and 72 h after the last training session. Part of these samples was separated, processed, and stored in a freezer at − 80 °C for further analysis of the metabolites. The other part was immediately used to assess glucose, insulin, and total cholesterol levels.

The fasting glucose concentrations were analyzed using an automatic chemistry analyzer and a commercially available kit (Laborlab, SP, Brazil). The insulin levels were determined by electrochemiluminescence, while total cholesterol was determined using commercially available kits (Roche Diagnostics GmbH, IN, USA). Insulin resistance (IR) was estimated by the homeostasis model assessment (HOMA-IR) using the formula: [(Fasting insulin (uU/mL) × Fasting glucose (mg/dL))/22.5]^49^.

### Cardiorespiratory and muscle strength assessments

A treadmill exercise protocol (Inbramed, model ATL, Brazil) was performed an analyzed breath-by-breath (K4b, Cosmed, Italy). The protocol was based on Libardi et al.^50^ and consisted of a 2-min warm-up at 4 km/h, followed by an increase of 0.3 km/h every 30 s until physical exhaustion. The treadmill was set at a 1% incline during the test. The recovery period consisted of 4 min, starting at 5 km/h, and reducing 1 km/h each minute. The peak oxygen consumption (VO_2peak_) was determined by the highest last 30 s mean value of consumption.

For the muscle strength assessment, the one-repetition maximum (1-RM) test was performed on the bench press and leg press equipments. For the warm-up, subjects performed ten repetitions at 50% of their estimated 1-RM, followed by 1 min of rest. After that, subjects performed three repetitions at 70% of their estimated 1-RM, followed by 3 min of recovery to start the test. A maximum of five attempts to find the highest weight for the 1RM was performed, increasing the weights progressively, with 3 min of rest between the attempts^51^.

### Nutritional assessment

All participants filled three 24-h dietary records (on different and non-consecutive days, being 2 weekdays and 1 weekend day), to obtain information on estimated total caloric intake and macronutrients, before and after the intervention. The adolescents had meetings with a nutritionist to avoid problems of under or over self-report in the dietary records, and to explain the importance of writing the truth in their records. The participants were instructed not to change their dietary habits during the study. The records were analyzed using the software Diet Pró, version 5i, by experienced nutritionists.

### Training protocol

The training protocol consisted of resistance training (RT) and aerobic training (AT) in the same session (Combined Training—CT), with a total of 60 min, three times a week, for 12 weeks. Only the TG performed the exercise training protocol. The CG was encouraged to maintain the same routine activities during the 12 weeks, without any change in the levels of physical activity and dietary patterns.

The RT was composed by six exercises (leg press 45°, leg extension machine, leg flexion machine, bench press, lat pulldown, and barbell biceps curl), with three sets of six to ten repetitions each and 1–2 min of rest between sets^11^. The exercises were alternated by segment (upper and lower). After the end of the RT, the volunteers performed 30 min of AT (walking or running), between 50 and 85% of VO_2peak_^11^. The adjustments of the training zone were previously described by Lopes et al.^11^. The training protocol started with 5 min of walking/jogging on a treadmill for warm-up.

### Metabolomics analysis

All procedures were previously described in Duft^12^. The sample preparation included the washing of the filters 3 kDa (Amicon Ultra) with H_2_0 Milli-Q and centrifugation at 20,817*g* (14,000 rpm in an *Eppendorf 5417R*) for 10 min at 4 °C. Then, the samples were centrifugated in the same rotation and temperature for 45 min. The filtered solution (200 µl) was transferred to a standard 5 mm NMR tube (Wilmad), and added 60 μL of phosphate buffer (pH 7.4 for the pH standardization), containing 0.5 mM of TMSP for internal chemical shift reference and 340 μL of Milli-Q H_2_O, completing a total of 600 μL in the NMR tube.

**Figure 5:**
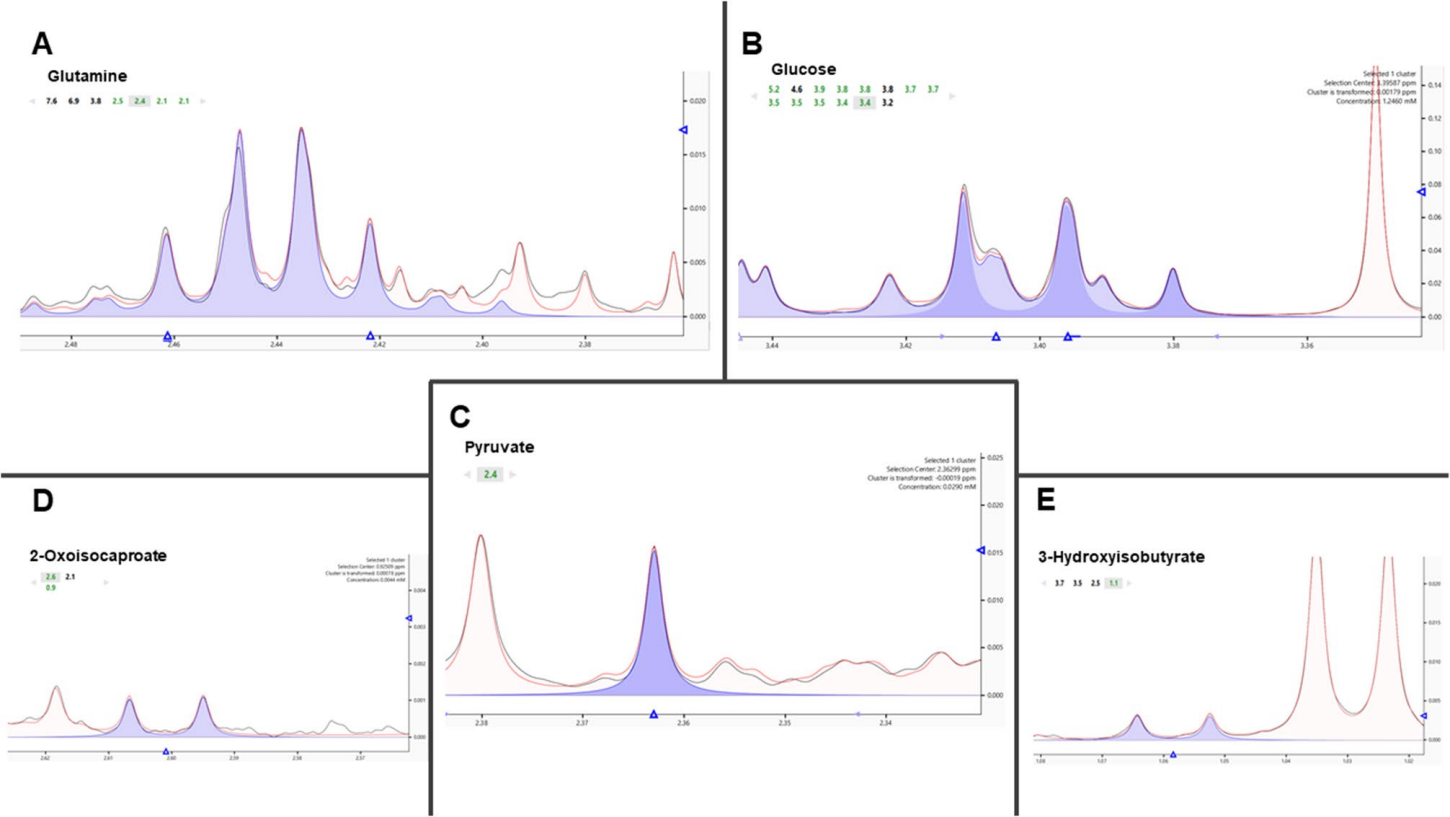
Representation of the ^1^H NMR spectrum region where the main metabolites were quantified. (**A**) Glutamine. (**B**) Glucose. (**C**) Pyruvate (**D**) 2-Oxoisocaproate and (**E**) 3-hydroxyisobutyrate.

The spectra acquisition was carried out by nuclear magnetic resonance spectroscopy (^1^H NMR) in a 600 MHz spectrometer (Varian Inova. Agilent Technologies. Santa Clara. CA), equipped with a triple cold probe. A total of 256 scans were collected with an acquisition time of 4 s and relaxation delay intervals between scans of 1.5 s. The temperature was maintained at 298 K (25 °C). After the acquisition, we realized the phase adjustment, baseline correction, spectral calibration, and quantification of metabolites, conducted by the software Chenomx NMR Suite 7.6 (Chenomx. Edmonton. AB. Canada)^12^ (Fig. 5).

### Statistical analysis

The multivariate data analysis was carried out by the MetaboAnalyst 4.0 software. Firstly, to decrease the variability of metabolites concentration values between and within groups, we calculated the fold change (FC, post divided by pre values) of all metabolites in order to increase the power analysis. For the normalization and to achieve greater symmetry between the data distribution curves, we used the auto-scaling technique for data standardization. The first multivariate analysis applied was orthogonal partial least square discriminant analysis (OPLS-DA), to verify the differences between the control and the training groups. The most important metabolites that explained the changes in the metabolic profile between groups were verified using S-PLOT graph. A visual inspection was made, followed by the selection of metabolites based on the cutoffs of p[1] and p(corr)[1] − 0.2 and 0.2, because the metabolites with the greatest influence on the cluster are located furthest away from the center of the S-plot^52^. Twenty metabolites were selected by these specifications. The quality and robustness of the model were reported by permutation tests (one hundred permutations p < 0.01) and cross-validation (R2Y = 0.986 and Q2 = 0.54), also conducted on MetaboAnalst 4.0.

After that, univariate analyses were conducted to compare specific changes in each metabolite and dependent variable resulting from the physical training. The data distribution and homogeneity of variances were tested by the Shapiro–Wilk test and the Levene's test. An independent t-test was conducted with pre moments to verify the necessity of an ANCOVA. For the analysis of dependent variables between and within the groups, the Repeated Measures ANOVA two-way was applied to assume as independent variables group (CG and TG) and moment (pre and post). Assumptions of sphericity were evaluated using the Mauchly’s test. Where sphericity was violated (p < 0.05), the Greenhouse–Geisser correction factor was applied. Whenever a significant F-value was obtained, a Bonferroni adjustment was performed for pairwise comparison purposes^12^. All p-values for main effects and group*moment interactions of metabolites were adjusted by the Benjamini–Hochberg false discovery rate rate method^53^. Finally, we used the Pearson and Spearman’s correlation coefficient to analyze the associations between significant changes in metabolites and changes in functional variables. These analyses were carried out using the IBM SPSS Statistics for Windows, Version 25.0. Armonk, NY. The level of statistical significance for all analyses was set at 5% (P < 0.05).

## Data Availability

The datasets generated during and/or analyzed during the current study are available from the corresponding author on reasonable request.
